# Impacts, and the Law That Governs Them

**Published:** 1879-04

**Authors:** W. H. Robinson

**Affiliations:** Suisun, Cal.


### IMPACTS, AND THE LAW THAT GOVERNS THEM.

BY W. H. ROBINSON, D. D. S., SUISUN, CAL.

The subtle little force that jumps from the mallet, strikes the plugger and makes the plugger strike the gold, we call an impact or blow. This impact may seem quite a simple manifestation of force, but it is an intricate manifestation—a result of many and causal factors.

The immediate formators of a plug are materials and force intelligently applied. The part, one of these factors, force plays in plug construction is my present theme. I have always recognized the importance of the materials, so much so that when we hear the term "plug" or "filling," the impression or idea we receive from these terms is more of the material than of the manipulative force that forms them. We detract nothing from the importance of the materials in plug construction; we only want to emphasize the fact that the formative force that changes materials into plugs, is equally an important factor in that completeness that tends toward perfection.

One fourth of an ounce of gold is material for a large plug, but it requires about one-thousand pounds of force to change this material into a plug. Our completed plug is, then, a compound of one-fourth ounce of gold and one thousand pounds of force. This force is not a neutral factor.

Some years ago the force in plug construction was applied through an instrument held in the hand, and was termed "hand pressure." Now it is applied in various ways and by numerous machines, some very simple, others quite complex and intricate. The manner of their application of force differing mainly from hand pressure in the sudden impact by which the force is expended on the plug. The simplest applier of force by impaction is the common hand mallet. The impact of the hand mallet differs mainly from the impacts of the automatic, pneumatic, electric and other machines, in being more immediately under the control of the operator. He can put more intelligence into the impact of the hand mallet than into the impact of any yet devised machine mallet. Force, as derived from the impact of the hand mallet is very variable. In the early days of mallets, the various and diverse effects of mallet impaction on the plug, tooth and adjacent tissue led to much discussion on the relative merits of different mallets, such as light, heavy, soft, hard, etc. I think no one will dispute this proposition, that the same plugger, moving with a given velocity, will always impart the same impact, no matter what the instrument or mode by which the plugger received its impetus. The application of this proposition must not lead us to suppose that the plugger itself, or the kind of motion under which it acts are not important.

The weight and motion of a plugger are the factors of its impact. Analyze these factors and we know just the kind of impact it will impart, *i.e.* just the kind of impact it receives modified by its own weight.

The factors of an impact being the weight and velocity of the plugger, the elimination of these tells us the kind of impact. The weight of any given plugger being always the same the character of the impact varies as the velocity.

Suppose we call the weight of a plugger two, and its velocity ten, its quantity of motion or momentum would be twenty, and when made to impinge on a plug, it would impart just that quantity of motion (twenty) to the plug. Another plugger with a velocity of two, and weight ten, has the same momentum, and imparts the same quantity of motion to the plug it impacts. But the effect produced on the plug at point of contact is widely different. If we suppose the impact of the latter plugger just sufficient to weld foil number two, then the impact of the former plugger, having five times the penetrating force would weld foil number ten, and the concussion or shock to the tooth and adjacent parts would be the same from both pluggers.

As to what kind of impactor is best for plug construction admits of a wide range of opinion, but I think all dentists will agree with me in this, that the impact that expends its major force directly on the gold, and imparts the least possible concussion or shock to the tooth and adjoining structures, is just the impact for plug, tooth, tissues and patient.

I admit that the sentient impressions of the patient and the observations of the operator are safe guides to follow, but until we comprehend, formulate and demonstrate the laws that govern mallet and plugger impaction as the rational deductions of these sensations and observations, we are groping our way in the shadowy land of empiricism.

Let me try some simple experiments and see what may be learned from them about the laws that govern impacts and impactors. I suspend from the ceiling of my laboratory a block of soft wood weighing about twenty-five pounds. I use a broad band or strap so to allow free oscillation without torsion. I proceed to drive into the suspended wood a few dozen common brass pins, using mallets or hammers ranging in weight from one-fourth ounce to one pound. I soon learn that heavy hammers are not good to put pins into suspended wood. As I descend the scale I find the lighter hammers work better, and with hammers from one-half to three ounces I succeed; above this weight they oscillate the wood too much and bend the pins, while the very light hammers do neither, but bur or rivet the pin heads. I take a series of say ten mallets, varying in weight from one-half ounce to eight ounces. Through a hole in their handles near the end I suspend these mallets on a horizontal wire, and so adjust them that I can have them impact the wood, or any object I wish on the wood. On the wood I adjust a piece of thick lead plate, and against this a plugger, so it will move as easily as when held in the fingers. I know the weight of each mallet, and measuring the distance I let it drop, so I can in this way ascertain the exact force of each blow. I assume as a unit of measure the force imparted by an impact from an ounce mallet let drop one foot, and call this an ounce blow. I now proceed to let each mallet drop one foot and strike the adjusted plugger against the lead on the suspended wood. I note the amount of oscillation, the impact each mallet produces, also that there is a series of impressions in the lead, decreasing in the same ratio as the weight of the mallets. I now regulate the drop of each mallet so that the impact of each will give the wood the same oscillation. The mallets weigh respectively one-half, one, two, three, four, five, six seven and eight ounces, and twenty-four, twelve, six, four, three, two and two-fifths, two, one and five-sevenths and one and one-half inches is the distance we let each drop, and represents its velocity. The weight of any given mallet into its velocity gives the momentum or quantity of motion, and now each mallet has a momentum of twelve.

If the mallets drop the distance given to each, and strike the adjusted plugger, each will impart the same quantity of motion to the wood, or cause it to oscillate the same distance. Now examine carefully the impress the plugger made in the lead under the impact of each mallet. Here we learn a fact important to the dental student, viz: while the oscillation of the wood was the same under the impact of each mallet, the impress in the lead is different in each case. The heaviest mallet making the lightest, and the lightest mallet making an impress just sixteen times as deep as the heaviest. The weight into the velocity gives the momentum or oscillation of the wood, but to ascertain the impress on the lead we take the weight into the square of the velocity. In this calculation I have not considered the weight of the plugger. How does the weight of the plugger effect the impact?

Again carefully re-adjust the drop of the mallets so that the striking force of all is the same. The impact of each makes the same impression in the lead, but the wood oscillates four and a half as much under the blow of the heavy as from the blow of the light mallet. I now use pluggers of various weights; first a two ounce one. When this receives an impact from a mallet of greater weight, it acquires the velocity of the striking mallet, and when that is less than the weight of the plugger, then the velocity the plugger receives decreases as the weight of the mallet. I now find the lead receives the deepest impresses from the impact of the medium weight mallets, because the larger ones were moving with the lesser velocity, and the weight of the plugger neutralized the impacts of the light mallets. Remember it is the momentum of the mallet that moves the plugger, and in this last case the mallets all had the same striking force. If I had put a small lead shot on the head of each plugger, the impact of each mallet would have flattened these shot equally; but in doing this the large mallet would move the plugger twelve times as far, or give it twelve times as much momentum as the impact from the light mallet. I now substitute a plugger weighing one-eighth ounce, and make the mallets impact it with the same striking force, and under each impact the lead receives the same impress, but the oscillation of the wood increases as the weight of the mallets, being twelve times as great under the heaviest as under the impact of the lightest mallet. I omit from these calculations the weight of the light plugger.

I make another experiment of more practical utility to the dentist. I substitute for the lead on the wood gold plate, surface prepared to cohere with foil, and upon this I proceed to weld some foil, say Williams' number twenty. The impact wanted in plug construction is the lightest that will properly bring in play the cohesive property of the gold. I find an impact of from one-fourth to one-half the force of what I called an ounce blow does this. Using an one ounce mallet and one eighth ounce plugger, the area of its point being equal to Varney's number two. An impact from the eight ounce mallet of the same striking force has the same welding force, but it oscillates the wood from eight to twelve times as much. I now try the welding power of the same impacts through a two ounce plugger. Through this the impact of the heavy mallet welds the gold, but it requires an impact from two to four times as forcible from the light mallet to weld gold through the heavy plugger as through the light one, and of course a corresponding increase in the oscillation of the wood or general concussion. I tabulate these results for comparison.

| WEIGHT OF MALLET. | WEIGHT OF PLUGGER. | VELOCITY OF MALLET. | VELOCITY OF PLUGGER. | STRIKING OR WELDING FORCE. | OSCILLATION OR CONCUSSION FORCE. |
|---|---|---|---|---|---|
| 1 ounce. | 2 ounce. | 4 | 2 | 8 | 4 |
| 1 " | $\frac{1}{8}$ " | 4 | $3\frac{7}{8}$ | $15\frac{1}{2}$ | $3\frac{7}{8}$ |
| 8 " | 2 " | 2 | $1\frac{1}{2}$ | 18 | 12 |
| 8 " | $\frac{1}{8}$ " | 2 | 2 | 32 | 16 |
| $\frac{1}{2}$ " | $\frac{1}{8}$ " | 16 | 16 | 32 | 2 |

I omit friction and intricate fractions, and assume perfect elasticity of pluggers and mallets. I do not claim absolute accuracy for these deductions, but an approximation that more thorough scientific research will confirm. The oscillation of the wood is a correct measure of the impact, also a correct measure of the concussion or jar the tooth and adjacent tissues receive in plug construction.

This concussion may not only be immediately painful to the patient and induce pathological conditions in the tooth and adjacent tissues, but concussion may, and often does, destroy the integrity of the plug itself. Take an ordinary molar after extraction, fill any ordinary shallow cavity in it carefully and thoroughly, using a light plugger and mallet, submit it to the severest leakage tests and it stands them. Now with mallet and plugger as heavy as some use, add some foil, or impact the plug for a minute or so as in ordinary consolidation, apply the test and the plug, in many cases, leaks. If the tooth structure is strong and retaining points extensive, the plug passes the ordeal unscathed, but the concussion of heavy impactors is one serious cause of leakage. The concussion and rebound between the plug and dentos destroys that perfect union between gold and tooth that is proof against leakage.

I can not speak "ex cathedra" as to the best materials for mallets. Habit has some influence over all of us. The disagreeable noise of some is specially objectionable. Mallets of perfectly elastic materials impart their force to the plugger, and rebound instantly. Soft, inelastic mallets have no rebound. Elastic mallets give their full velocity to the plugger, but no weight. Inelastic mallets divide their velocity with the plugger and add their weight to it. The handle of the mallet is perhaps of more importance than the head. The short, stiff handle of the average dental depot mallet is an abomination. An impact from one of them has for its weight factor, mallet, handle and hand of operator, even when the operator holds ever so loosely to avoid hand weight.

Due proportions between heads and handles of all such tools as hammers and mallets is a fact carefully studied by the skillful workman. Take two mallets of equal weight, handles ten inches long, one having the ordinary stiff, heavy handle; the other same size where held by hand, but whalebone or some very elastic material, and so small about two inches from the head that it springs and bends readily in ordinary usage. Now take a few old porcelain teeth and rivet down the pins, carefully noting the working of each hammer. We break fewer teeth with the limber handle. The rebound of hammers or mallets as the case may be is different and so is the impact. The handles of all Dental mallets should be so limber as to spring readily in ordinary usage. Such woods as elm, hickory, etc., are good mallet material. They avoid the disagreeable noises of steel, ivory, etc., and have about the desirable degree of elasticity. To those accustomed to heavy pluggers and mallets, when first using light ones they seem to be doing but little effective execution; but if they experiment with both and accurately note results they will soon be convinced what they have got rid of, is not effective execution but noise and concussion.

The impact of all our automatic mallets have too much weight and too little velocity. Consequently too much concussion. The impacts of most of our Electric, Pneumatic and engine impactors, have the same defects but not to the same extent; indeed some of these are tending toward a proper adjustment of the weight and velocity elements in their impact. The main objection to them is, their impacts are not directly under the control of the operator.

For that high type of manipulative execution that delicate operations on frail teeth require, rained muscle obeying intelligence educated to the highest point of executive discernment, will always be the best motor; and the common hand mallet is now and I think will continue to be the truest exponent of this motor.

I make a few practical deductions.

The best impact for dental operations is that which has the weight element at the lowest practicable point and the velocity element at the highest practicable point.

Pluggers should have the least weight, that will give strength sufficient to convey the impact from mallet to plug, or from one-eigth to one-fourth ounces.

The weight of mallets should be the least that will give sufficient impetus to the plugger; or from three-fourths to one and one-half ounces, and with handles so limber as to spring readily in ordinary use and from ten to twelve inches long.

