# Correction

**Published:** 1879-05

**Authors:** 


### Editorial.

### CORRECTION.

On page 147 of the April number of the REGISTER is the name of Dr. W. H. Robinson, D. D. S., author of the article there printed. Now the Doctor objects to the D. D. S., not that he dislikes the title, but he says it does not belong to him. I think those who read the article will conceed that he ought to have it. He is not the author or the owner of the aforesaid D. D. S.

